# Efficient One-Pot Synthesis of 3,4-Dihydropyrimidin-2(1*H*)-ones via a Three-Component Biginelli Reaction

**DOI:** 10.3390/molecules26123753

**Published:** 2021-06-20

**Authors:** Giovanna Bosica, Fiona Cachia, Riccardo De Nittis, Nicole Mariotti

**Affiliations:** Department of Chemistry, University of Malta, 2080 Msida, Malta; fiona.cachia.14@um.edu.mt (F.C.); riccardo.denittis.17@um.edu.mt (R.D.N.); nicole.mariotti@unito.it (N.M.)

**Keywords:** Biginelli reaction, multicomponent reaction, heterogeneous catalysis, solvent-free, supported heteropoly acid, one-pot

## Abstract

Multicomponent reactions are considered to be of increasing importance as time progresses due to the economic and environmental advantages such strategies entail. The three-component Biginelli reaction involves the combination of an aldehyde, a β-ketoester and urea to produce 3,4-dihydropyrimidin-2(1*H*)-ones, also known as DHPMs. The synthesis of these products is highly important due to their myriad of medicinal properties, amongst them acting as calcium channel blockers and antihypertensive and anti-inflammatory agents. In this study, silicotungstic acid supported on Ambelyst-15 was used as a heterogeneous catalyst for the Biginelli reaction under solventless conditions. Electron-poor aromatic aldehydes gave the best results. Sterically hindered β-ketoesters resulted in lower reaction yields. The reaction was carried out under heterogeneous catalysis to allow easy recovery of the product from the reaction mixture and recycling of the catalyst. The heterogeneity of the reaction was confirmed by carrying out a hot filtration test.

## 1. Introduction

3,4-Dihydropyrimidin-2(1*H*)-ones (DHPMs) are heterocyclic compounds with a pyrimidine moiety in the ring nucleus which, in recent decades, have aroused interest in medicinal chemistry due to their versatile biological activity. DHPMs possess a broad range of pharmacological activities and are widely used in pharmaceutical applications [1,2]. The variety of pharmacological aspects associated with DHPM derivatives includes being potential anticancer, anti-inflammatory, antioxidant and antimicrobial agents as well as having antimalarial and antitubercular effects. Additionally, DHPMs can be found in numerous alkaloids in several marine sources. Such alkaloids contribute to the synthesis of biologically active natural products, which are crucial in the medicinal field. Certain alkaloids have shown cytotoxicities against a number of tumor cell lines, as well as antifungal activity against Candida albicans and antiviral activity against the herpes simplex virus [3]. Furthermore, it was found that Batzelladine A and B, two naturally occurring marine alkaloids, include a DHPM unit in their structure. Since these alkaloids are believed to inhibit the binding of HIV gp-120 to CD_4_ cells, DHPMs could potentially be considered for AIDS therapy [4].

DHPMs are usually synthesized through a multicomponent reaction (MCR) [5] in which three or more starting materials react to form a product, where basically all or most of the atoms contribute to the newly formed product. MCRs aim to increase the efficiency of a standard chemical synthesis, usually by targeting, to improve economical and chemical means for production, as well as to strive for eco-friendly objectives. MCRs are not usually intended to create new pathways, but rather seek alternative strategies which provide additional advantages to the process and its products. Such advantages include lessening the waste generation, time, energy, solvent use and human effort attributed to chemical reactions [5].

**Scheme 1:**
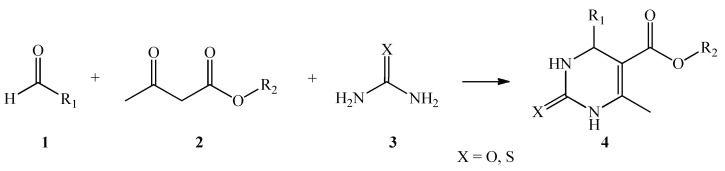
The Biginelli reaction.

The Biginelli reaction is a strategic methodology for the production of dihydropyrimidinone (X=O) and dihydropyrimidinthione (X=S) derivatives (**4**) (DHMPs) [6]. It involves a condensation reaction between an aldehyde (**1**), a β-ketoester (**2**) and urea (**3**, X=O), as shown in Scheme 1.

The Biginelli reaction is typically catalyzed by a Brønsted or a Lewis acid, although a Brønsted base-catalyzed version of the reaction has also been reported with an interesting mechanistic investigation [5].

Pietro Biginelli first reported this reaction in the late nineteenth century but it is in the late twentieth century that new synthetic methodologies were studied and new applications for DHPMs discovered [6]. The first studies employed the use of protic catalysts, such as hydrochloric acid and phosphoric acid. A wide variety of Lewis acid catalysts have also been studied, including: Yb(OTf)_3,_ LiBr, B(OH)_3_, CuI, Fe(NO_3_)_3_·9H_2_O, HBF_4,_ PhB(OH)_2_, InBr_3_ ZrOCl_2_·8H_2_O, ZrCl_4_ [7,8,9,10]. However, some of the reported methods suffer from drawbacks derived from the product isolation procedure and environmental pollution. Moreover, in the case of sensitively functionalized aromatic and aliphatic aldehydes, the strongly acidic conditions and prolonged time of heating required in the original Biginelli reaction do not provide DHPM derivatives in high yields (only 20–40%) [11]. It is in recent years that the interest in heterogeneous catalysts has grown and the replacement of conventional toxic and polluting Brønsted and Lewis acid catalysts by solid acid heterogeneous catalysts has achieved an important role in the synthesis of 3,4-dihydropyrimidinones [11]. Thus, many examples of solid acid catalysts have been reported in the literature for performing the Biginelli reaction with variable success, such as: acid activated montmorillonite clay, cobalt supported on alumina, molybdenum supported on silica, FeCl_3_ supported on polyaniline [12,13,14,15]. It has been reported that in the Biginelli reaction, a catalyst not only provides better yields and reaction times, but is also fundamental in improving the selectivity towards one reaction pathway. Considering that there are three debated mechanisms proposed for this MCR, i.e., iminium, enamine or Knoevenagel mechanisms, although the iminium mechanism is supported by stronger evidence, and the number of possible intermediates, it can be appreciated how fundamental the investigation of more efficient catalysts and greener reaction conditions of the Biginelli MCR is [4,5,16].

Following our ongoing study on the development of greener methodologies for multicomponent reactions [17], we decided to investigate the possibility of discovering new, alternative catalysts which could give comparable yields to those found in the literature, whilst being environmentally friendly, considering that the Biginelli products possess a wide range of applications, especially in the medical field due to the easy synthesis of heterocyclic compounds. It was also our aim to optimize the reaction conditions and to perform the reaction over a variety of aldehydes and β-ketoesters, in order to exploit its versatility over different substrates.

A one-pot multicomponent approach as a route providing a high atom economy was used in combination with heterogeneous catalysis, since it offers a greener alternative to a homogeneous one, with the solid insoluble catalyst having the advantage of being easily removed from the reaction mixture via filtration and ideally recycled, unlike a soluble one [18,19]. Moreover, a solvent-free reaction condition to reduce the amount of waste and hazardous chemicals from solvent is preferred. 

Silicotungstic acid (WSi) anchored to Amberlyst-15 beads (A-15) [20] was found to be a valuable heterogeneous catalyst able to promote the synthesis of a variety of DHPMs through a one-pot Biginelli reaction under solvent-free conditions. The catalyst is both easy and safe to prepare, as well as recoverable and recyclable. 

## 2. Results

### 2.1. Catalyst Screening and Condition Optimization

**Scheme 2:**
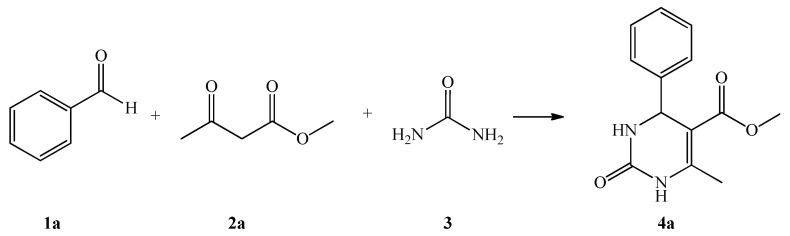
The Biginelli model reaction under study: benzaldehyde (**1a**), methyl acetoacetate (**2a**) and urea (**3**).

According to literature, the reaction has been shown to work best and most efficiently under acidic conditions since such conditions enhance the selectivity, so various catalysts, mainly acidic, were tested for the model Biginelli reaction shown in Scheme 2. 

The catalyst screening was performed using 0.05 g/mmol of catalyst in 10 mL of ethyl acetate, with a temperature of 88 °C, since visible refluxing was observed upon setting the oil bath to this particular temperature. The initial reagent ratio used was 1:1:1, benzaldehyde (**1a**): methyl acetoacetate (**2a**): urea (**3**) on a 5 mmol scale. The results obtained are shown in Table 1. All heteropolyacids used were in powder form.

The results shown in Table 1 include four divisions of catalysts, these being (i) Lewis acids on basic support, using CuI on Amberlyst-A21, (ii) clay catalysts, using montmorillonite clays, (iii) Lewis acids on neutral support, using CuI on neutral alumina and (iv) heteropolyacids. From all these categories, heteropolyacids have proven to be the best catalysts overall, whereas basic conditions did not produce any result (entry 2). The third best catalyst group overall was the montmorillonite clay catalysts, where montmorillonite was an aluminosilicate with a general formula of Al_2_Si_4_O_10_(OH)_2_·*n*H_2_O. The second most effective catalyst group, as seen from entry 5 of Table 1, was simple neutral catalysis using CuI on neutral alumina Cu(I), which acted as a Lewis acid, and alumina was expected to conform towards the reaction environment due to its amphoteric properties. The best catalyst in Table 1 is clearly silicotungstic acid supported on Amberlyst-15 (WSi/A15) (entry 9), giving a result of 70% yield. Although WSi supported on alumina (WSi/Al_2_O_3_) and phosphotungstic acid supported on silica (PW/SiO_2_) gave similar promising results (entries 7 and 8), due to the powder nature of these supports, our interest focused on WSi loaded onto Amberlyst-15 beads, which are more easily recoverable owing to their physical nature.

The optimization of the process was then carried out on the model reaction with benzaldehyde, methyl acetoacetate and urea, on a 5 mmol scale, using WSi/A15 in different quantities and different loadings, as reported in Table 2. The reaction was tested under different temperatures, reactant ratios and reaction times and under solvent-free conditions. At first, we performed the reaction using 10 mL of ethanol at 88 °C under refluxing conditions, since we observed a reduction in reaction time and a yield increase compared to the initial reaction in ethyl acetate (entries 3, 8 and 9) and then in solvent-free conditions, with a few drops of ethanol being added occasionally when solidification occurred, in order to allow unhindered stirring, in an oil bath kept at 92 °C. The reaction was monitored through the use of thin layer chromatography using a solvent mixture of 5:5 hexane:ethyl acetate. Increasing the amount of catalyst loading did have a positive effect on the reaction yields. The molar ratios of reagents were altered by increasing the urea in the model reaction. The best result (yield = 82%, entry 12) was obtained, at a reactant ratio of 1:1:1.2, using 0.05 g/mmol of WSi/A-15 at a higher loading of 40% *w*/*w* under stirring for 4.5 h. The pure product was dried under vacuum overnight. In contrast, homogeneous WSi gave a good result (entry 15) but could not be retrieved after the reaction. Moreover, when the reaction was performed using A15, only the yield decreased whilst reaction time increased drastically (entry 17). The model reaction under the optimal selected conditions was then tried on a scale three times higher obtaining similar results.

### 2.2. Substrate Screening

The optimal reaction conditions chosen satisfied the green protocol we were aiming for. Therefore, from this stage, we moved on to the next one by changing the substrates to explore the versatility of the developed method. Different aldehydes and β-ketoesters were employed in order to see how the yield and reaction times would vary compared to those of the model reaction, as shown in Table 3. Selectivity was promising even for the other substrates used. Various aromatic substituted benzaldehydes were used and all gave similar results with generally minimal time and yield deviations from the model reaction. Each reaction was carried out with the aldehyde, β-ketoester and urea present in a ratio of 1:1:1.2 on a 5 mmol scale, using 0.05 g/mmol of 40% *w*/*w* WSi/A-15 and each reaction was set at an oil bath temperature of 92 °C. 

According to the iminium mechanism, the most accepted one, the rate-determining step was taken to be the initial nucleophilic attack of urea on the aldehydic electron-deficient carbon [4]. Hence, the more electrophilic the carbon becomes, the more susceptible it is to attack. Negative mesomeric effects, along with negative inductive effects, signify electron withdrawal from the rest of the ring. Positive mesomeric and inductive effects, on the other hand, induce electron donation to the ring, which would signify a reduction in the electrophilicity exhibited by the carbon in the aldehyde functional group, hindering nucleophilic attack by urea. The results of negative inductive effects can be appreciated when comparing the yields and reaction times of those products obtained when using 4-fluorobenzaldehyde (**1c**), 4-chlorobenzaldehyde (**1d**), 4-bromobenzaldehyde (**1b**) and 4-nitrobenzaldehyde (**1i**) as substrates. Meanwhile, both 4-hydroxybenzaldehydes (**1h**) and 4-methoxybenzaldehyde (**1j**) are found to be in the middle of the spectrum with regard to their reaction times (6 h and 8 h, respectively). This is in line with their higher positive mesomeric effects.

From the results obtained, it can be noted that the choice of the β-ketoester does not affect the yield of the reaction as much as aldehydes do. Since the addition of the β-ketoester is not part of the rate-determining step, it does not affect the rate of the reaction to a great extent. The use of both methyl acetoacetate (**2a**) and ethyl acetoacetate (**2b**) resulted in appreciable yields, although, with the latter, a higher yield was obtained. A very appreciable yield was obtained with benzyl acetoacetate (**2c**), while more sterically hindered β-ketoesters, such as isobutyl acetoacetate (**2e**), resulted in a lower reaction yield. When using an even more sterically hindered substituent in R_2_, like *t-*butyl, the yields were very low, so these trials were abandoned.

### 2.3. Hot Filtration Test

To ascertain whether the reaction was proceeding under heterogeneous conditions, a hot filtration test was performed to determine whether leaching of the acid from the resin support occurred. No significant leaching of silicotungstic acid was observed from the resin support since, upon removing the catalyst, product formation did not occur, in accordance with what was previously reported about this catalyst [20]. The Biginelli synthesis carried out was therefore proved to proceed under heterogeneous conditions. 

### 2.4. Catalyst Recycling Test

**Figure 1:**
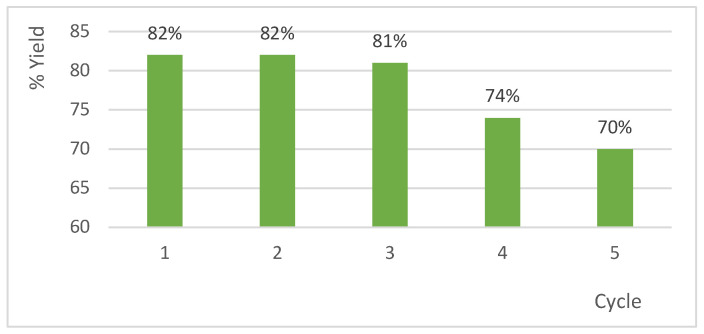
Percent (%) yield for every cycle run in the catalyst recycling test.

The model reaction between benzaldehyde, ethyl acetoacetate and urea was repeated for up to five cycles with the same recycled catalyst, with the yield decreasing from 82 to 70% (Figure 1). Between each cycle, the catalyst was filtered and then dried in an oven at 100 °C for 12 h.

### 2.5. Green Metrics

Green chemistry incorporates the efficient utilization of materials and the elimination of waste production and use of hazardous substances. The first green metrics were introduced in the early 1990s in an attempt to quantify the greenness and the sustainability of the chemical process. Such calculations involve the determination of the environmental (E) factor as well as calculation of the atom economy [26].

By definition, the E-factor (E stands for environmental) is the ratio of the total weight of the waste generated over the weight of product produced. The waste generated is the difference between the mass of reactants and products. The E-factor is therefore ideal to quantify the amount of waste generated and identify the resource intensity of the reaction. The closer the E-factor value is to zero, the less waste is generated, meaning that the production can be considered sustainable and with lower environmental impacts. The E-factor of the model reaction (**4a**) is equal to (details are reported in the Supplementary Materials file):

(1) $$E-\mathrm{factor}=\frac{\mathrm{mass}\;\mathrm{of}\;\mathrm{waste}\;}{\mathrm{mass}\;\mathrm{of}\;\mathrm{product}\;}=\frac{(1.471\;-\;1.009)\;}{1.009\;}=0.46$$

Most of the experimented reactions have an E-factor value close to zero, as a direct result of recoverable catalyst and solvent-free conditions, meaning that the Biginelli synthesis and its variations produce little waste, and can therefore be regarded as a green and sustainable process.

Another important parameter to be considered in green chemistry metrics is the atom economy. It represents the percentage of reagents incorporated into the final product. It is calculated by dividing the relative molecular mass (RMM) of the product by the sum of the RMM of all the reagents used and multiplying this ratio by 100.

The atom economy for the model reaction (**4a**) is equal to:

(2) $$\mathrm{AE}=\frac{{\mathrm{RMM}\;}_{\mathrm{products}}}{\sum^{\text{​}}{\mathrm{RMM}}_{\;\mathrm{starting}\;\mathrm{materials}}}\;\times \;100\;=\frac{246.27}{\left(106.12+116.12+60.06\right)}\;\times \;100=\frac{246.27}{282.30}\;\times \;100=87\%\;$$

High values for atom economy were obtained for all the experimental reactions, meaning efficient incorporation of the reagents into the final product occurred.

## 3. Materials and Methods

### 3.1. General

All commercially available chemicals were purchased from Aldrich (St. Louis, MO, USA) and used without further purification. IR spectra were recorded on an IR Affinity-1 FTIR spectrometer (Shimadzu, Kyoto, Japan) calibrated against a 1602 cm^−1^ polystyrene absorbance spectrum. Samples were analyzed as thin films in between sodium chloride discs. The ^1^H NMR spectra were recorded on an Avance III HD^®^ NMR spectrometer (Bruker, Coventry, England), equipped with an Ascend 500 11.75 Tesla superconducting magnet, operating at 500.13 MHz for ^1^H, and a multinuclear 5 mm PABBO probe (Bruker, Coventry, England). Samples were dissolved in the deuterated solvent specified in the section on the analytical information. Melting points of products were measured using a Stuart^®^ SMP11 melting point determination apparatus fitted with a mercury thermometer. Reactions were monitored using TLC plates composed of silica on PET with a fluorescent indicator and GC on a Shimadzu GC-2010 plus gas chromatograph equipped with a flame ionization detector and HiCap 5 GC column with dimensions of 0.32 mm (internal diameter) × 30 m (length) × 0.25 mm (film thickness), using nitrogen as carrier gas. Plates were observed under a UV lamp at a wavelength of 254 nm before staining in an iodine-saturated chamber.

### 3.2. Overall Method 

A double-necked round-bottom flask was secured to a Liebig condenser and 5 mmol of benzaldehyde, 5 mmol of methyl acetoacetate, 6 mmol of urea and 250 mg of WSi/A-15 (40% *w*/*w*) were added to it. A few drops (two microliters) of ethanol were added to initiate the reaction and avoid solidification. The reaction mixture was stirred in an oil bath at 92 °C. The reaction progress was monitored through thin layer chromatography (5:5 = Hex:EtOAc). The reaction mixture was filtered with hot ethanol and the catalyst recovered. The excess solvent was removed by using a rotary evaporator. The crude product was dried in a desiccator and weighed. Recrystallization of the crude product was carried out using ethanol as a solvent. The pure product was then dried in a desiccator and weighed.

### 3.3. Hot Filtration Test

The optimized model reaction was repeated. After 20 min from the beginning of the reaction, it was ensured that product formation occurred by running a TLC plate. After that, the catalyst was removed by filtration, using ethanol solvent for the workup. The reaction mixture was concentrated using a rotary evaporator and then left under reflux conditions for 2.5 h. If leaching of the catalyst occurred from the resin support, product formation would have been expected. After that, the mixture was filtered to check if any crystals of the product could be recovered.

### 3.4. Catalyst Recycling Test

The catalyst recycling test was carried out repeating the model reaction between benzaldehyde, ethyl acetoacetate and urea for a given number of cycles, using the same recycled catalyst, until a substantial drop in the yield was observed. Between each cycle, the catalyst was filtered and left to dry in the oven overnight at 100 °C. The reaction was carried out at a temperature of 92 °C under solventless conditions. The reagents were present in a ratio of 5:5:6 mmol benzaldehyde:ethyl acetoacetate:urea. The reaction time of 4.5 h was maintained for all cycles.

### 3.5. Analytical Information

*5-methoxycarbonyl-6-methyl-4-phenyl-3,4-dihydropyrimidin-2(1H)-one* (**4a**) [27]. White solid (1.009 g). Melting point: 211 °C; ^1^H-NMR (500 MHz, CDCl_3_): δ 7.86 (s, 1H, NH), 5.64 (s, 1H, NH), 7.31 (m, 5H, aromatic protons), 5.40 (d, 1H, *J* = 3.40 Hz, CH), 3.62 (s, 3H, CH_3_), 2.35 (s, 3H, CH_3_); IR (NaCl) *v*: 3331, 2923, 2853, 1771, 1697, 1653, 1506, 1457, 1239, 1236, 1094, 756, 698 cm^−1^.

*5-(ethoxycarbonyl)-6-methyl-4-phenyl-3,4-dihydropyrimidin-2(1H)-one* (**4b**) [28]. Yellow solid (1.145 g). Melting point: 204 °C; ^1^H-NMR (500 MHz, CDCl_3_): δ 7.47 (s, 1H, NH), 5.51 (s, 1H, NH), 7.31 (m, 5H, aromatic protons), 5.41 (d, 1H, *J* = 3.36 Hz, CH), 4.07 (m, 1H, CH_2_), 2.35 (s, 3H, CH_3_), 1.16 (t, 3H, *J* = 7.05 Hz, OCH_2_CH_3_); IR (NaCl) *v*: 3239, 3117, 3026, 2953, 2922, 2870, 2853, 1724, 1648, 1636, 1457, 1221, 956, 879, 758, 722, 667 cm^−1^.

*4-(4-bromophenyl)-5-(methoxycarbonyl)-6-methyl-3,4-dihydropyrimidin-2(1H)-one* (**4c**) [29]. Yellow solid (1.170 g). Melting point: 213.5 °C; ^1^H-NMR (500 MHz, DMSO-*d*_6_): δ 9.26 (s, 1H, NH), 7.78 (s, 1H, NH), 7.5–7.0 (m, 4H, ArH), 5.12 (d, 1H, *J* = 3.53 Hz, CH), 3.53 (s, 3H, CH_3_), 2.25 (s, 3H, CH_3_); IR (NaCl) *v*: 3364, 3217, 3099, 2953, 2923, 1716, 1685, 1635, 1457, 1228, 959, 765, 722, 688, 668 cm^−1^. 

*4-(4-fluorophenyl)-5-(methoxycarbonyl)-6-methy-3,4-dihydropyrimidin-2(1H)-one* (**4d**) [30]. Yellow solid (1.294 g). Melting point: 197 °C; ^1^H-NMR (500 MHz, DMSO-*d*_6_): δ 2.25 (s, 3H, -CH_3_), 3.53 (s, 3H, -CH_3_), 5.15 (d, 1H), 7.26 (m, 4H), 7.75 (s, 1H), 9.23 (s, 1H); IR (NaCl) *v*: 3219, 3084, 3013, 2953, 2923, 1733, 1653, 1558, 1457, 1376, 1219, 940, 843, 769, 722 cm^−1^.

*4-(4-chlorophenyl)-5-(methoxycarbonyl)-6-methy-3,4-dihydropyrimidin-2(1H)-one* (**4e**) [27]. White solid (1.291 g). Melting point: 195 °C. ^1^H-NMR (500 MHz, DMSO-*d*_6_): δ 9.25 (s, 1H, NH), 7.78 (s, 1H, NH), 7.40–7.23 (m, 4H, *J* = 8.40 Hz, aromatic protons), 5.14 (d, 1H, J = 3.42 Hz, CH), 3.53 (s, 3H, OCH_3_), 2.24 (s, 3H, CH_3_). IR (KBr) *v:* 3323, 3110, 1724, 1697, 1647, 1491, 1461, 781 cm^−1^.

*4-(2-chlorophenyl)-5-(methoxycarbonyl)-6-methy-3,4-dihydropyrimidin-2(1H)-one* (**4f**) [31]. Yellow solid (0.701 g). Melting point: 250 °C; ^1^H-NMR (500 MHz, DMSO-*d*_6_): δ 9.32 (s, 1H, NH), 7.72 (s, 1H, NH), 7.44–7.29 (m, 4H, *J* = 8.40 Hz, aromatic protons), 5.65 (d, 1H, *J* = 3.40 Hz, CH), 3.49 (s, 3H, OCH_3_), 2.34 (s, 3H, CH_3_); IR (NaCl) *v*: 3208, 3134, 3004, 2959, 2854, 1717, 1648, 1558, 1458, 1224, 1091, 959, 818, 761 cm^−1^. 

*5-benzyloxycarbonyl-6-methyl-4-phenyl-3,4-dihydropyrimidin-2(1H)-one* (**4g**) [32]. Yellow solid (1.241 g). Melting point: 170 °C; ^1^H-NMR (500 MHz, DMSO-*d*_6_): δ 9.25 (s, 1H, NH), 7.74 (s, 1H, NH), 7.32–7.15 (m, 10H, aromatic protons), 5.19 (d, 1H, *J* = 3.41 Hz, CH), 5.02 (q, 2H, *J* = 7.2, CH_2_O), 2.28 (s, 3H, CH_3_); IR (NaCl) *v*: 3254, 3128, 3005, 2959, 2924, 2868, 2853, 1717, 1653, 1636, 1457, 1219, 962, 773, 721, 649 cm^−1^. 

*Benzyl 4-(4-fluorophenyl)-1,2,3,4-tetrahydro-6-methyl-2-oxopyrimidine-5-carboxylate* (**4h**) [33]. Yellow solid (1.105 g). Melting point: 181 °C; ^1^H-NMR (500 MHz, DMSO-*d*_6_): δ 9.29 (s, 1H, NH), 7.76 (s, 1 H, NH), 7.30–7.10 (m, 9H, HAr), 5.19 (d, *J* = 2.7 Hz, 1H, CH_2_), 5.02 (q, 2H, *J* = 7.2, CH_2_O), 2.28 (s, 3H, -CH_3_); IR (NaCl) *v*: 3218, 3117, 3007, 2955, 2920, 2868, 2852, 1732, 1682, 1635, 1455, 1376, 1297, 960, 723, 649 cm^−1^.

*4-(2,4-dichlorophenyl)-5-(methoxycarbonyl)-6-methyl-3,4-dihydropyrimidin-2(1H)-one* (**4i**) [34]. Yellow solid (0.598 g). Melting point: 253 °C; ^1^H-NMR (500 MHz, DMSO-*d*_6_): δ 9.33 (s, 1H, NH), 7.75 (s, 1H, NH), 7.50 (d, 1H, *J* = 1.59 Hz, HAr), 7.43–7.40 (dd, 1H, *J*1 = 9.00 Hz, *J*2 = 2.02 Hz, HAr), 7.33 (d, 1H, *J* = 7.86 Hz, HAr), 5.59 (d, 1H, *J* = 3.02 Hz, CH), 3.46 (s, 3H, -CH_3_), 2.30 (s, 3H, -CH_3_); IR (NaCl) *v*: 3213, 3113, 3018, 2954, 2924, 1717, 1647, 1558, 1457, 1229, 1094, 968, 819, 745, 656 cm^−1^.

*Ethyl 2-oxo-4-phenyl-6-trifluoromethyl-1,2,3,4-tetrahydro-pyrimidine-5-carboxylate* (**4j**) [35]. Yellow solid (0.580 g). Melting point: 203 °C; ^1^H-NMR (500 MHz, CDCl_3_): δ 7.64 (s, 1H, NH), 7.23 (s, 1H, NH), 7.39–7.33 (m, 5H, aromatic protons), 4.79 (d, 1H, *J* = 2.39 Hz, CH), 3.78 (m, 2H, CH_2_), 0.81 (s, 3H, CH_3_); IR (NaCl) *v*: 3221, 3107, 3009, 2955, 2924, 2870, 2855, 1717, 1653, 1457, 1377, 1186, 935, 775, 722, 700 cm^−1^.

*5-isobutyloxycarbonyl-6-methyl-4-phenyl-3,4-dihydropyrimidin-2(1H)-one* (**4k**) [36]. Yellow solid (0.590 g). Melting point: 143 °C; ^1^H-NMR (500 MHz, DMSO-*d*_6_): δ 9.19 (s, 1H, NH), 7.72 (s, 1H, NH), 7.34–7.31 (m, 2H, aromatic protons), 7.26–7.23 (m, 3H, aromatic protons), 5.16 (d, 1H, *J* = 3.53 Hz, CH), 3.78–3.69 (d, 2H, *J* = 6.69 Hz, CH_2_OCO) 2.29 (s, 3H, CH_3_), 1.8–1.72 (m, 1H, CH); 0.76–0.74 (m, 6H, (CH_3_)_2_); IR (NaCl) *v*: 3255, 3122, 3030, 2955, 2923, 2870, 2853, 1699, 1652, 1636, 1457, 1220, 982, 772, 722, 669 cm^−1^. 

*Methyl 6-methyl-2-oxo-4-p-tolyl-1,2,3,4-tetrahydropyrimidine-5-carboxylate* (**4l**) [37]. White solid (0.858 g). Melting point: 211 °C. ^1^H-NMR (500 MHz, DMSO-*d*_6_): δ 9.16 (s, 1H, NH), 7.68 (s, 1H, NH), 7.11 (s, 4H, aromatic protons), 5.10 (d, 1H, *J* = 3.32 Hz, CH), 3.52 (s, 3H, OCH_3_), 2.26 (s, 3H, CH_3_), 2.24 (s, 3H, CH_3_); IR (KBr) *v:* 3240, 3107, 1709, 1697, 1651 cm^−1^.

*Ethyl 6-methyl-2-oxo-4-(p-tolyl)-1,2,3,4-tetrahydropyrimidine-5-carboxylate* (**4m**) [38]. White solid (1.083 g). Melting point: 205 °C. ^1^H-NMR (500 MHz, DMSO-*d*_6_): δ 9.13 (s, 1H, NH), 7.66 (s, 1H, NH), 7.11 (s, 4H, aromatic protons), 5.10 (d, 1H, *J* = 3.32 Hz, CH), 4.05–4.02 (q, 2H, *J* = 7.07 Hz, OCH2), 2.26 (s, 3H, CH_3_), 1.99 (s, 3H, CH_3_), 1.09 (t, 3H, OCH_2_CH_3_, *J* = 1.10 Hz); IR (KBr) *v:*3326, 3175, 1647, 1221, 1051 cm^−1^.

*5-Methoxycarbonyl-4-(4-hydroxyphenyl)-6-methyl-3,4-dihydropyrimidin-2(1H)-one* (**4n**) [39]. Pink solid (0.852 g). Melting point: 240 °C. ^1^H-NMR (500 MHz, DMSO-*d*_6_): δ 9.31 (s, 1H, OH), 9.12 (s, 1H, NH), 7.61 (s, 1H, NH), 7.03–7.01 (d, 2H, *J* = 8.49 Hz, aromatic protons), 6.69–6.67 (d, 2H, *J* = 8.59 Hz, aromatic protons), 5.03 (d, 1H, *J* = 3.43 Hz, CH), 3.52 (s, 3H, OCH3), 2.23 (s, 3H, CH3); IR (KBr) *v:* 3225, 1678, 1636 cm^−1^.

*Ethyl-1,2,3,4-tetrahydro-6-methyl-4-(4-nitrophenyl)-2-oxopyrimidine-5-carboxylate* (**4o**) [40]. Yellow solid (1.312 g). Melting point: 200 °C. ^1^H-NMR (500 MHz, DMSO-*d*_6_): δ 9.34 (s, 1H, NH), 8.22 (d, 2H, *J* = 8.79 Hz, aromatic protons), 7.88 (s, 1H, NH), 7.51–7.50 (d, 2H, *J* = 8.72 Hz, aromatic protons), 5.28 (d, 1H, *J* = 3.38 Hz, CH), 4.01–3.97 (q, 2H, *J* = 7.00, 7.10 Hz, CH_2_), 2.27 (s, 3H, CH_3_), 1.10 (t, 3H, *J* = 7.05 Hz, OCH_3_); IR (KBr) *v:* 3233, 3129, 1730, 1697, 1643, 1522, 1348 cm^−1^.

*Methyl 4-(4-methoxyphenyl)-6-methyl-2-oxo-1,2,3,4-tetrahydropyrimidine-5-carboxylate* (**4p**) [41]. Orange solid (1.132 g). Melting point: 185 °C. ^1^H-NMR (500 MHz, DMSO-*d*_6_): δ 9.16 (s, 1H, NH), 7.67 (s, 1H, NH), 7.15–7.13 (d, 2H, *J* = 8.81 Hz, aromatic protons), 6.88–6.86 (d, 2H, *J* = 8.68 Hz, aromatic protons), 5.09 (d, 1H, *J* = 3.40 Hz, CH), 3.72 (s, 3H, ph-OCH3), 3.52 (s, 3H, OCH_3_), 2.24 (s, 3H, CH_3_); IR (KBr) *v:* 3248, 3113, 1709, 1647 cm^−1^.

*Methyl 4-(furan-2-yl)-6-methyl-2-oxo-1,2,3,4-tetrahydropyrimidine-5-carboxylate* (**4q**) [41]. Brown solid (0.885 g). Melting point: 200 °C. ^1^H-NMR (500 MHz, DMSO-*d*_6_): δ 9.25 (s, 1H, NH), 7.76 (s, 1H, NH), 7.55 (s, 1H, furyl-H), 6.35–6.34 (m, 1H, furyl-H), 6.09 (d, 1H, *J* = 3.22 Hz, furyl-H), 5.20 (d, 1H, *J* = 3.56 Hz, CH), 3.57 (s, 3H, OCH_3_), 2.23 (s, 3H, CH_3_); IR (KBr) *v:* 3318, 3119, 2955, 1701, 1670, 1636, 1505, 1437, 1381, 1340, 1277, 1238 cm^−1^.

## 4. Conclusions

The one-pot multicomponent Biginelli reaction for the synthesis of dihydropyrimidinone derivatives was performed under green heterogeneous and neat conditions with a range of aldehydes, β-ketoesters and urea in a ratio of 1:1:1.5, in the presence of 0.05 g/mmol of 40% *w*/*w* silicotungstic acid on an Amberlyst-15 catalyst, which is easy, safe and environmentally benign to prepare, fully recoverable and reusable for up to five runs. A high atom economy of 87% and a low E-factor of 0.95 highlight the greenness of the procedure. More importantly, SiW/A15 was able to catalyze a wide range of reactions involving different aromatic aldehydes to give products in good to excellent yields with electron-poor aldehydes, performing much better than electron-rich or bulky ones, whereas sterically hindered β-ketoesters resulted in lower reaction yields and longer reaction times.

## Figures and Tables

**Table 1:** Catalyst screening for the model reaction involving benzaldehyde (**1a**), methyl acetoacetate (**2a**) and urea (**3**).

Entry ^a^	Catalyst	Yield (%) ^b^	Reaction Time (hours)
1	Montmorillonite K30	62	14
2	CuI–Amberlyst 21 ^c^ (1.59 mmol/g)	No yield	20
3	Fe(II) Montmorillonite K10 ^d^	16	20
4	Zn(II) Montmorillonite K10 ^d^	26	20
5	CuI–Alumina ^e^ (1.39 mmol/g)	60	20
6	40% PW/Al_2_O_3_ ^f^	30	11
7	40% PW/SiO_2_ ^f^	68	20
8	40% WSi/Al_2_O_3_ ^f^	66	20
9	30%WSi/A15 ^g^	70	20

^a^ Reaction carried out in ethyl acetate at 88 °C using 0.05 g/mmol of catalyst and a ratio of 1:1:1 of reactants benzaldehyde (**1a**), methyl acetoacetate (**2a**) and urea (**3**), respectively. ^b^ Yields of pure isolated products. ^c^ Catalyst was prepared according to method reported in [21]. ^d^ Catalyst was prepared according to method reported in [22]. ^e^ Catalyst was prepared according to method reported in [23]. ^f^ Catalyst was prepared according to method reported in [24]. ^g^ Catalyst was prepared according to method reported in [25].

**Table 2:** Condition optimization using WSi/A15 catalyst.

Entry	% *w*/*w* WSi on A15	Reactant Ratio ^a^	Mass of Catalyst (g/mmol of Benzaldehyde) [WSi Molar Percentage] (mmol %)	Yield (%) ^b^ [Time (hrs)]
1 ^c^ 2 ^c^ 3 ^c^ 4 ^d^ 5 ^e^ 6 ^e^ 7 ^e^ 8 ^e^ 9 ^e^	30%	1:1:1.2 1:1:1.2	0.05 [0.52] 0.035 [0.36]	58 [4.5] 45 [4]
		1:1:1	0.05 [0.52]	60 [4.5]
		1:1:1.2 1:1:1.2 1:1:1.2 1:1:1.2 1:1:1 1:1:1.2	0.05 [0.52] 0.05 [0.52] 0.05 [0.52] 0.035 [0.36] 0.05 [0.52] 0.05 [0.52]	52 [4.5] 76 [4.5] 69 [1.5] 61 [1.5] 62 [1.5] 79 [20]
10 ^e^ 11 ^e^	38%	1:1:1.2	0.068 [0.9]	75 [4.5]
		1:1:1.2	0.095 [1.25]	78 [3.5]
12 ^e^ 13 ^e^ 14 ^e^	40%	1:1:1.2 1:1:1.2	0.05 [0.69] 0.05 [0.69]	82 [4.5] 65 [2.5]
		1:1:1.2	0.05 [0.69]	66 [3.5]
15 ^f^	Tungstosilicic acid hydrate	1:1:1 1:1:1	0.012 [0.4] 0.012 [0.4] 0.0012 [0.04] 0.010 [0.35] 0.012 [0.4]	47 [6] 64 [20] 13 [6] 36 [6] 78 [12]
16 ^g^	-	1:1:1.2	- -	0 [24]
17 ^h^	A15	1:1:1.2 1:1:1	0.05 -	54 [6] 63 [16]

^a^ Molar ratio of benzaldehyde:methyl acetoacetate:urea. ^b^ Yields of pure isolated products. ^c^ Reaction carried out in ethanol at 88 °C on a 5 mmol scale. ^d^ Reaction carried out in ethanol at 96 °C on a 5 mmol scale. ^e^ Reactions carried out solventless under neat conditions at 92 °C on a 5 mmol scale. ^f^ Reaction performed in neat but homogeneous conditions using 0.4 mmol % of unsupported WSi (0.012 g) at 92 °C. ^g^ Reaction performed in the absence of any catalyst in neat conditions at 92 °C. ^h^ Reaction performed in the presence of 0.05 g/mmol of A15 only (previously dried at 105 °C) at 92 °C on a 5 mmol scale.

**Table 3:** Yields and reaction times for the Biginelli reaction involving aldehydes (**1a-l**), β-ketoesters (**2a-c**) and urea (**3**).

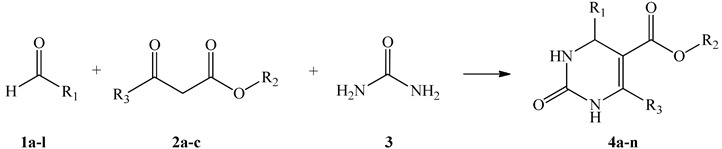					
**Product ^a^**	**R_1_**	**R_2_**	**R_3_**	**Yield *(%)*** **^b^**	**Reaction Time (h)**
**4a**	** 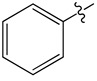 ** **1a**	CH_3_	CH_3_	82	5
		**2a**			
**4b**	** 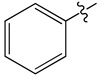 ** **1a**	CH_3_CH_2_	CH_3_	88 ^c^	4.5
		**2b**			
**4c**	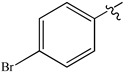 **1b**	CH_3_	CH_3_	72	6
		**2a**			
**4d**	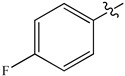 **1c**	CH_3_	CH_3_	98	4
		**2a**			
**4e**	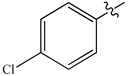 **1d**	CH_3_	CH_3_	92	22
		**2a**			
**4f**	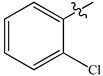 **1e**	CH_3_	CH_3_	50	5
		**2a**			
**4g**	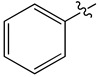 **1a**	C_6_H_5_CH_2_	CH_3_	77	6
		**2c**			
**4h**	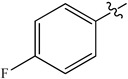 **1c**	C_6_H_5_CH_2_	CH_3_	65	6
		**2c**			
**4i**	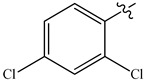 **1f**	CH_3_	CH_3_	38	5
		**2a**			
**4j**	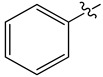 **1a**	CH_3_CH_2_	CF_3_	37	11
		**2d**			
**4k**	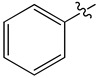 **1a**	(CH_3_)_2_CHCH_2_	CH_3_	41	8
		**2e**			
**4l**	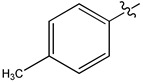 **1g**	CH_3_	CH_3_	66	6
		**2a**			
**4m**	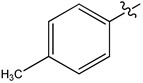 **1g**	CH_3_CH_2_	CH_3_	79	7
		**2b**			
**4n**	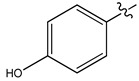 **1h**	CH_3_	CH_3_	65 ^c^	6
		**2a**			
**4o**	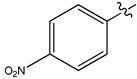 **1i**	CH_3_CH_2_	CH_3_	86 ^c^	23
		**2b**			
**4p**	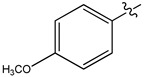 **1j**	CH_3_	CH_3_	82	8
		**2a**			
**4q**	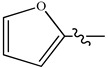 **1k**	CH_3_	CH_3_	75	4.5
		**2a**			

^a^ Reactions were all performed in neat conditions on a 5 mmol scale in the presence of 0.05 g/mmol of 40% *w*/*w* WSi/A-15 and a ratio of 1:1:1.2 of reactants, aldehydes, β-ketoesters and urea, respectively. ^b^ Isolated product. ^c^ Average value of two different trials.

## Data Availability

The data presented in this study are available in the article or supplementary materials.

## Supplementary Materials

The spectroscopic data of all products are available online.

## Sample Availability

Samples of the products are available from the corresponding author.
